# The spontaneously produced lysogenic prophage phi456 promotes bacterial resistance to adverse environments and enhances the colonization ability of avian pathogenic *Escherichia coli* strain DE456

**DOI:** 10.1186/s13567-024-01292-z

**Published:** 2024-03-26

**Authors:** Dezhi Li, Wei Liang, Zhiqiang Huang, Wenwen Ma, Qing Liu

**Affiliations:** 1 School of Health Science and Engineering, University of Shanghai for Science and Technology, Shanghai, China; 2 The Fourth Affiliated Hospital of Guangxi Medical University, Liuzhou, China; 3 Key Laboratory of Animal Bacteriology, Ministry of Agriculture, College of Veterinary Medicine, Nanjing Agricultural University, Nanjing, China

**Keywords:** Prophage, colonization, biofilm, environmental stress, avian pathogenic *E. coli*

## Abstract

In the last decade, prophages that possess the ability of lysogenic transformation have become increasingly significant. Their transfer and subsequent activity in the host have a significant impact on the evolution of bacteria. Here, we investigate the role of prophage phi456 with high spontaneous induction in the bacterial genome of Avian pathogenic *Escherichia coli* (APEC) DE456. The phage particles, phi456, that were released from DE456 were isolated, purified, and sequenced. Additionally, phage particles were no longer observed either during normal growth or induced by nalidixic acid in DE456Δphi456. This indicated that the released phage particles from DE456 were only phi456. We demonstrated that phi456 contributed to biofilm formation through spontaneous induction of the accompanying increase in the eDNA content. The survival ability of DE456Δphi456 was decreased in avian macrophage HD11 under oxidative stress and acidic conditions. This is likely due to a decrease in the transcription levels of three crucial genes—*rpoS*, *katE*, and *oxyR*—which are needed to help the bacteria adapt to and survive in adverse environments. It has been observed through animal experiments that the presence of phi456 in the DE456 genome enhances colonization ability in vivo. Additionally, the number of type I fimbriae in DE456Δphi456 was observed to be reduced under transmission electron microscopy when compared to the wild-type strain. The qRT-PCR results indicated that the expression levels of the subunit of I fimbriae (*fimA*) and its apical adhesin (*fimH*) were significantly lower in DE456Δphi456. Therefore, it can be concluded that phi456 plays a crucial role in helping bacterial hosts survive in unfavorable conditions and enhancing the colonization ability in DE456.

**Supplementary Information:**

The online version contains supplementary material available at 10.1186/s13567-024-01292-z.

## Introduction

The most abundant biological entity on Earth is bacteriophages (phages). They have an estimated global population of 10^31^ [1]. Phages are viruses that infect bacteria and can undergo lytic and lysogenic life cycles based on their genetics and interaction with the bacterial host. After infecting bacteria, a lytic phage's nucleic acid enters the cell, using the host's transcription and translation mechanism to produce phage-related proteins. Finally, the bacteria is dissolved to release the newly formed phages and complete the infection cycle [2]. In contrast, temperate phages integrate their DNA into the bacterial genome, becoming a prophage [3]. Phage-like elements have been found in nearly all bacterial genomes, regardless of whether they are pathogenic or not, that have been sequenced. It has been observed that prophage sequences can constitute up to 20% of the bacterial genome [3]. However, the role of many prophages in bacterial biological properties is ill defined.

As prophage replication follows the bacterial genome, it can be understood that prophage propagation depends on the host bacterium to some extent. The presence of prophage in the bacterial genome is undoubtedly an extra metabolic burden on bacteria. Therefore, if a prophage does not confer any fitness to the host, the bacteria carrying the prophage will be at a disadvantage in the competition and eventually disappear with natural selection [4, 5].

It has been demonstrated that some prophages exert a profound and beneficial influence on cellular physiology. For example, in terms of biofilms, prophage SV1 in *streptococcus pneumoniae* [6], phi458 in APEC [7], and Muso1, Muso2, and Lambda So in *Shewanella oneidensis* [8] have been shown to enhance biofilm formation by spontaneous induction and increased eDNA release. In terms of resisting adverse environments, it has been extensively researched in *E. coli* K-12 BW25113 that removing all nine prophages from BW25113 not only decreased cell growth but also reduced the survival rate in adverse environments such as osmolarity, H_2_O_2_, and acid [9]. Moreover, it is essential to note that prophages have the potential to impact the virulence of bacteria [3, 10]. Toxins are biological poisons that help bacteria invade and damage tissues. Some toxins are directly encoded by prophages, such as the diphtheria toxin produced by *Corynebacterium diphtheriae* [11], Shiga toxins found in *E. coli* O157:H7 [12], botulinum toxin from *Clostridium botulinum*, SpoE effector protein of *Salmonella enterica subsp. enterica* serovar Typhimurium [13], and cholera toxin produced by *Vibrio cholerae* [14]. On the other hand, the virulence factors of bacteria can also be regulated by prophages [15]. In these studies, the regulation of prophages on bacterial motility has been widely reported [16, 17]. Bacterial motility is essential for bacterial pathogenicity, as it facilitates surface colonization, host tissue invasion, and nutrient acquisition [18].

Extraintestinal pathogenic *E. coli* (ExPEC) are facultative pathogens, which include neonatal meningitis *E. coli* (NMEC), uropathogenic *E. coli* (UPEC), sepsis-associated *E. coli* (SEPEC), and avian pathogenic *E. coli* (APEC). APEC is accountable for avian species' extra-intestinal infections, commonly called colibacillosis. Colibacillosis is a leading cause of illness and death in poultry, contributing to various disease conditions such as bloodstream infection, head swelling, heart lining inflammation, sinus infection, inflammation of the navel area, abdominal cavity infection, and air sac inflammation. APEC can act as a primary or secondary pathogen in these cases [19]. APEC shares phylogenetic overlap with neonatal meningitis *Escherichia coli* (NMEC) and shares many virulence factors, such as type I fimbriae, invasins, and protectins [20]. Additionally, it has also been demonstrated that APEC can cause meningitis or bacteremia in mice, similar to NMEC [21]. Thus, APEC strains are believed to serve as a reservoir of virulence genes for NMEC, thereby enhancing its zoonotic potential.

Our previous study found that when grown in an LB medium or infected with chickens, DE456 could release phage particles [7]. However, the role of prophages that can switch to the lytic life cycle in DE456 remains unknown. Hence, the released particles from DE456 were isolated and sequenced. Subsequently, a corresponding prophage deletion mutant strain was constructed to further investigate the effect of prophages in DE456.

## Materials and methods

### Bacterial strains and culture conditions

The bacterial strains and prophage used in this study are listed in Table 1. The APEC strain DE456 used in this study was isolated from a duck with clinical neurological signs, and was identified as serogroup O2. The O2 serogroup is a prevalent serogroup among APEC strains [22]. The strains were cultured in Luria–Bertani (LB, Sigma-Aldrich, USA) medium under aerobic conditions at 37 ℃.

**Table 1:** **Bacterial strains and phages used in this study**

Strain or phage	Description	Source
DE456	Wild-type APEC strain isolated from a duck	This study
DE456Δphi456	Strain cure of prophage phi456	This study
Phi456	Prophage induced from DE456	This study
MC1061	The indicator strain of phage phi456	[7]

### Phage purification and propagation

A purified single colony of DE456 isolate was cultured in LB at 37 ℃. Once the OD_600_ of the culture reached approximately 1.0, nalidixic acid (Sigma-Aldrich, USA) with a final concentration of 1 µg/mL was added. Then, the culture was centrifugated at 5000 × *g* for 5 min, and the supernatant was collected and filtrated with a 0.22-μm-pore-size membrane filter. The above filtrates were screened directly for phage particles using *E. coli* K-12 derivative strain MC1061 as an indicator [23], and subsequently propagated and purified by a double-layer agar method.

### Transmission electron microscopy (TEM)

The morphological characteristics of DE456 and DE456Δphi456 and isolated phages were examined using TEM as described previously [24] with some modifications. To prepare the phage sample, a plate with plenty of abundant plaques whose edges were visible was added with 3 mL of sterilized double deionized H_2_O; To prepare the bacteria samples, each bacterial suspension was taken to streak onto LB agar plates, which were then incubated at 37 for 12 h. Bacterial colonies were suspended in 3 mL of sterilized double deionized H_2_O. After 10 min of incubation and gentle shaking, the supernatant was subjected to centrifugation at 5000 × *g* for 10 min. Subsequently, the supernatant was adsorbed onto carbon-coated copper grids and followed by staining with phosphotungstic acid (PTA [pH 7.0], Sigma-Aldrich, USA) for 5 s. The morphologies of the phages and bacteria were captured using a Hitachi H7650 electron microscope in a random manner by a colleague.

### Induction rates in different environments

To determine the excision rates of phi456 under different temperatures, individual DE456 colonies were selected and cultured overnight at either 28 ℃ or 42 ℃ for 12 h. To assess the excision rates of phi456 when interacting with cells, the determination method is similar to the adhesion assay and HD-11 intracellular survival assay, which will be detailed in later related assays, respectively.

The bacterial DNA was extracted from the cells grown under various environmental conditions and used as a template for quantitative real-time PCR. The number of bacterial genomes without phi456 was quantified using primers (Δphi456-F/R) flanking of phi456. Due to the size of phi456, PCR products are present only when phi456 is removed from the DE456 genome [25]. Real-time PCR experiments were carried out using the AceQ qPCR SYBR Green Master Mix (Vazyme, Nanjing, China) reagents on a Biorad CFX apparatus (Hercules, CA, USA). The reaction mixture included primers with a final concentration of 0.3 µM and appropriately diluted DNA solutions. For amplification, a 2-step process was employed. Firstly, pre-denaturation was carried out at 95 °C for 5 min. This was followed by 40 cycles of 95 °C for 10 s and 60 °C for 30 s. Finally, elongation was performed at 95 °C for 15 s, 60 °C for 1 min, and 95 °C for 15 s. To confirm the amplification specificity, the melting curves of PCR products were analyzed at the end of the reaction. The results were analyzed using CFX Manager v. 3.1 software (Biorad, Hercules, CA, USA). The target sequence was quantified by normalizing to the reference gene *purA*. The assays were performed in triplicate and repeated five times. The primers used are listed in Table 2.

**Table 2:** **Primers used in this study**

Primer	Sequence (5′–3′)	Target gene
16S-F	GCGGAGCATGCGGATTA	16S
16S-R	AACGTGCTGGCAACATAGGG	
Δphi456-F	TCAGGCCTATCCAGTAC	Deletion of phi456
Δphi456-R	CGGTATCGTATGACTGA	
qPCR-phi456-F	TCGTGATAACACACGCAAC	Circular phi456
qPCR-phi456-R	TGAACCTGAAACAATGGAC	
RT-*fimH*-F	CTTATGGCGGCGTGTTATCT	*fimH*
RT-*fimH*-R	CGGCTTATCCGTTCTCGAATTA	
RT-*fimA*-F	GTTGTTCTGTCGGCTCTGTC	*fimA*
RT-*fimA*-R	TAAAGTGAACGGTCCCACCA	
RT-*ropS*-F	AGAGTAACTTGCGTCTGGTGGTAAA	*ropS*
RT-*ropS*-R	ATAGTACGGGTTTGGTTCATAATCG	
RT-*katE*-F	GATCTTCTCGATCCAACCAAAC	*katE*
RT-*katE*-R	CACCAAGACGACTGATTTGTGT	
RT-*oxyR*-F	TGAGGTGAAAGTCCTTAAAGAGATG	*oxyR*
RT-*oxyR*-R	GTCTGTGCTTCATGCAGATACATT	
TLR3-F	AACGGAGTTGCAAGGGAATG	TLR3
TLR3-R	ATCAGGTCAGGTGCTCTCCC	
TLR4-F	GCTCCTGGCTAGGACTCTGAT	TLR4
TLR4-R	GGCTAGCAGGAAAGGGTGTG	
TLR9-F	CCTCTCTCCAAGCCCTACCT	TLR9
TLR9-R	CAGTCCTGGTTCTGAAGCCT	
TNF-α-F	TCTCCTTCCTGATCGTGGCA	TNF-α
TNF-α-R	GGAGAGTGGATGAAGGCTGG	
TNF-β-F	CAGGAACCCAAGCATCCACC	TNF-β
TNF-β-R	AGGAAGTGGGCACTGAACAA	
β-Actin-F	GCTGTCCCTGTATGCCTC	β-Actin
β-Actin-R	AGATGTCAGCGCACCAC	

### Extraction of phage DNA and sequencing

Purified phage particles were obtained through PEG-8000 precipitation (Shanghai Sangon, China), followed by DNA isolation from the phage particles via the phenol/chloroform method, as previously described [26]. About 1 μg DNA was sheared into 400–500 bp fragments using a Covaris M220 Focused Acoustic Shearer following manufacture’s protocol. Illumina sequencing libraries were prepared from the sheared fragments. The prepared libraries then were used for paired-end Illumina sequencing (2 × 150 bp) on an Illumina NovaSeq 6000 machine in Shanghai Winnerbio Technology Co., Ltd. (Shanghai, China). The raw sequenced reads underwent low-quality read filtering and were subsequently assembled using Unicycler v 0.43 to get a full-length phage genome. Software tRNAscan-SE (version 2.0.8) was used for tRNA detecting [27]. ORFs were predicted by PHASTER [28], and the virulence genes were screened by VirulenceFinder.

### Isolation of DE456Δphi456

The assays were performed as previously described with some modifications [29]. To increase the excision rate of phi456, a single colony of DE456 was cultured in LB broth at 42 ℃ for 12 h, and then the culture was diluted and spread on LB agar plates. The plates were incubated at 37 °C until individual colonies became visible, and then two thousand colonies were picked into LB broth in 96-well plates. After cultured overnight at 37 ℃, about 1 µL bacterial culture was utilized as a template for screening the strain that phi456 lost by detecting the absence of predicted PCR product using primers flanking of phi456. 16S rRNA gene sequencing and metagenomic sequencing were used to further confirm the phi456 deletion strain was derived from DE456. The strain DE456Δphi456 was obtained and stored at -80 ℃. The primers used are listed in Table 2.

### Biofilm assay

As previously described [30], biofilms were measured by crystal violet staining. In brief, log-phase bacterial cultures were adjusted to an OD_600_ of 0.6, followed by diluting 1:100 in LB broth, and 200 µL of each culture was dispensed into 96-well polystyrene microplates (Costar; Corning, USA). The sterile LB broth was used as the control wells. After incubation at 37 ℃ for 36 h, the plates were washed twice with phosphate buffer saline (PBS, Shanghai Sangon, China) to remove unattached planktonic cells. The attached cells in each well were allowed to air dry and subsequently fixed by incubation with 200 μL of methanol per well for 15 min. After air drying, the biofilm biomass was stained with 1% (w/v) crystal violet (Sigma-Aldrich, USA) for 20 min. Following washing with double-distilled water and drying, 200 μL of 95% ethanol was added to each well and incubated for 15 min. The absorbance of 600 nm of each well was determined. The samples were assayed in triplicate, with LB medium serving as the negative control. The experiments were replicated thrice.

The DNase control group was inoculated with cells in LB media supplemented with DNase I (Shanghai Sangon, China) at a final concentration of 100 μg/mL [29]. After biofilm formation, the supernatant after centrifugation was filtered through a 0.22 μm filter, and the release of phage particles phi456 during biofilm formation was measured by the double-layer plate method with MC1061 as the indicator strain.

### Resistance to environmental stress

The environmental stress resistance assays were conducted in 1.5 mL tubes, following the previously described protocol [9]. In brief, the bacteria in the logarithmic growth phase were collected and washed twice with ice-cold PBS, then diluted the bacterial concentration to 5 × 10^7^ CFU/mL. Approximately 20 μL of diluted bacterial suspension was added to 180 μL of medium containing 30 mM H_2_O_2_ (Shanghai Sangon, China), acidic (pH 3) (Shanghai Sangon, China), 2.4 M NaCl (Shanghai Sangon, China) and PBS (Shanghai Sangon, China) (65 °C). After different incubation periods, the surviving bacteria were quantified by serial dilution on LB agar plates. The experiments were performed in triplicate and repeated four times.

### Adhesion assay

The bacterial adhesion ability was measured by the DF-1 cell line as previously described [31]. DF-1 cell monolayers derived from chicken embryos were cultured in 24-well plates containing Dulbecco's modified Eagle's medium (DMEM, Gibco, USA) supplemented with 10% fetal bovine serum under a humidified atmosphere containing 5% CO_2_ at 37 °C for 16 h. The bacteria were subsequently subjected to centrifugation at 5000 × *g* for 10 min and then washed with DMEM. Then, the bacterial suspension was added to the wells with a multiplicity of infection (MOI) of 100. After incubation for two hours under conditions of 37 °C and 5% CO_2_, adherent bacteria were washed with ice-cold PBS, while non-adherent (planktonic) bacteria were discarded. The DF-1 cells were lysed with 0.5% Triton X-100 and the number of adherent bacteria was determined by plating on MacConkey agar (Shanghai Sangon, China) plates. The experiment was conducted in five biological replicates.

### Analysis of phage cytotoxicity

The DF-1-cell monolayers derived from chicken embryo fibroblasts were prepared according to the previously described [32]. Similar to the adhesion experiment, DF-1 cells were grown in 24-well cell culture plates (Costar; Corning, USA) with DMEM (Gibco, USA, containing 10% FBS) at 37 °C until they reached confluence. Then, approximately 200 μL of trypsin was added to each well. After incubating for 3–5 min, the cells were washed twice with DMEM and re-suspended in 2 mL of DMEM. DF-1 cell suspension was mixed with 50 μL purified phage and incubated in a 24-well cell culture plate at 37 °C under 5% CO_2_ humidity for 24 h. DMEM and SM were used as negative controls. Following trypsinization, cell viability was assessed using Trypan blue assays. Specifically, 4 μL of 0.4% Trypan blue solution in PBS was added to the cell suspensions and the cells were immediately assessed under an inverted light microscope using five squares of view per sample. The assays were performed in triplicate and repeated at least three times.

### Analysis of phage effect on toll-like receptors and tumor necrosis factors

This assay was conducted using the chicken macrophage cell line HD-11. The HD-11 cells were allowed to adhere to a 24-well cell culture plate (Costar; Corning, USA) at a density of 2 × 10^5^ cells/mL in RPMI 1640 (Gibco, USA) (containing 10% fetal bovine serum and 2 mM L-glutamine) at 37 ℃ with 5% CO_2_ for overnight. Subsequently, 200 μL of trypsin was added to each well, followed by an incubation period of 3–5 min. The cells were then subjected to two washes with DMEM and re-suspended in a volume of 2 mL of DMEM. A mixture containing HD-11 suspension and 50 μL purified phage (1 × 10^6^ PFU/mL) was placed in a 24-well cell culture plate and incubated at 37 °C under humid conditions with 5% CO_2_ for 24 h. The DMEM served as the negative control group. qRT-PCR was used to determine the transcriptional level of toll-like receptors and tumor necrosis factor. The specific primers (Table 2) were designed using primer-BLAST based on reference sequences in NCBI database and were synthesized by Shanghai Sangon Biotech. The assays were performed in triplicate and repeated three times.

### Intracellular macrophage survival

This assay was conducted using the chicken macrophage cell line HD-11. The HD-11 cells were allowed to adhere to a 24-well cell culture plate at a density of 2 × 10^5^ cells/mL in RPMI 1640 (containing 10% fetal bovine serum and 2 mM L-glutamine) at 37 ℃ with 5% CO_2_ for overnight. The two strains (DE456 and DE456Δphi456) in the logarithmic growth stage (OD_600_ = 0.6) were collected and washed twice with PBS. Then, the bacterial suspension was added to the wells with a multiplicity of infection (MOI) of 20. After incubating for 1 h, the cells were washed twice with PBS, and the medium was replaced with the RPMI (containing 50 μg/mL gentamicin). Incubation continued for another hour to eliminate the extracellular bacteria on the surface, and the cells were washed twice with pre-warmed RPMI. The cells were lysed using 1% triton X-100 (Shanghai Sangon, China), and the number of bacteria that survived in HD-11 cells was measured by performing serial dilutions at a fourfold ratio followed by plating on MacConkey agar plates. The assays were performed in triplicate and repeated four times.

### Assessment of systemic infection in chickens

Thirty-two chickens (7-day-old) were randomly divided into four groups. The two strains (DE456 and DE456Δphi456) were collected in the logarithmic growth stage and washed twice with ice-cold PBS, then diluted the bacterial concentration to 1 × 10^8^ CFU/mL with ice-cold PBS. Purified phage phi456 was cultured by the double-layer agar method. Subsequently, SM buffer (0.05 M Tris–HCl, pH 7.5, 0.1 M NaCl, 0.017 M MgSO4, 0.01% gelatin) was added to the plates, which were full of plaques, and incubated 4 ℃ for 12 h. The SM buffer was collected, and the bacteria were filtered out with a 0.22-μm filter followed by ultrafiltration with a 100 kD ultrafiltration tube to remove residual bacterial endotoxins. After the determination of the concentration of the harvest phages by the double-layer method, the phage concentration was diluted to 10^7^ PFU/mL. Two groups of chickens were challenged intratracheally with 0.1 mL of either DE456 (1 × 10^7^ CFU) or DE456Δphi456 (1 × 10^7^ CFU). The third group of chickens was challenged intratracheally with a mixture of DE456Δphi456 (1 × 10^7^ CFU) and phi456 (1 × 10^6^ PFU). The remaining eight chickens, serving as the control group, were intratracheally administered with an equivalent volume of PBS. At 24 h post-infection (hpi), the chickens were euthanized and samples from left lung, heart and left liver were collected. After grinding and dilution, the bacterial loads in these organs were estimated by counting on MAC agar plates. Each sample was measured three times and averaged.

### RNA isolation and qRT-PCR

The DE456 and DE456Δphi456 strains were cultured until reaching an optical density of 0.6. Subsequently, bacterial RNA was extracted by an E.Z.N.A. bacterial RNA isolation kit (Omega, USA) and was converted into cDNA through a HiScript II QRT Supermix (Vazyme Biotech, China). Real-time PCR experiments were carried out using the AceQ qPCR SYBR Green Master Mix (Vazyme, Nanjing, China) reagents on a Biorad CFX apparatus (Hercules, CA, USA). The reaction mixture included primers with a final concentration of 0.3 µM and appropriately diluted DNA solutions. For amplification, a 2-step process was employed. Firstly, pre-denaturation was carried out at 95 °C for 5 min. This was followed by 40 cycles of 95 °C for 10 s and 60 °C for 30 s. Finally, elongation was performed at 95 °C for 15 s, 60 °C for 1 min, and 95 °C for 15 s. To confirm the amplification specificity, the melting curves of PCR products were analyzed at the end of the reaction. The results were analyzed using CFX Manager v. 3.1 software (Biorad, Hercules, CA, USA). The endogenous reference gene *DnaE* was selected as an internal control, and the 2^−ΔΔCt^ method was employed to determine the relative expression level of mRNA. The assays were performed in triplicate and repeated three times. The primers used are listed in Table 2.

### Ethics statement

The animal study protocol was approved by the Ethical Committee for Animal Experiments of Nanjing Agricultural University (SYXK(SU)2017-0007), Nanjing, China.

### Statistical analyses

All assays were performed at least in triplicate. The data from this study were analyzed by GraphPad Prism (GraphPad Software, La Jolla, CA, USA). The in vivo colonization data was analyzed using Mann–Whitney *U*-tests. Student’s *t*-test was used to analyze the remaining data; A *P* value below 0.05 was considered significant.

### Accession number

The sequence of phi456 was submitted to the GenBank database (accession number OR339795).

## Results

### Genome analysis

**Figure 1:**
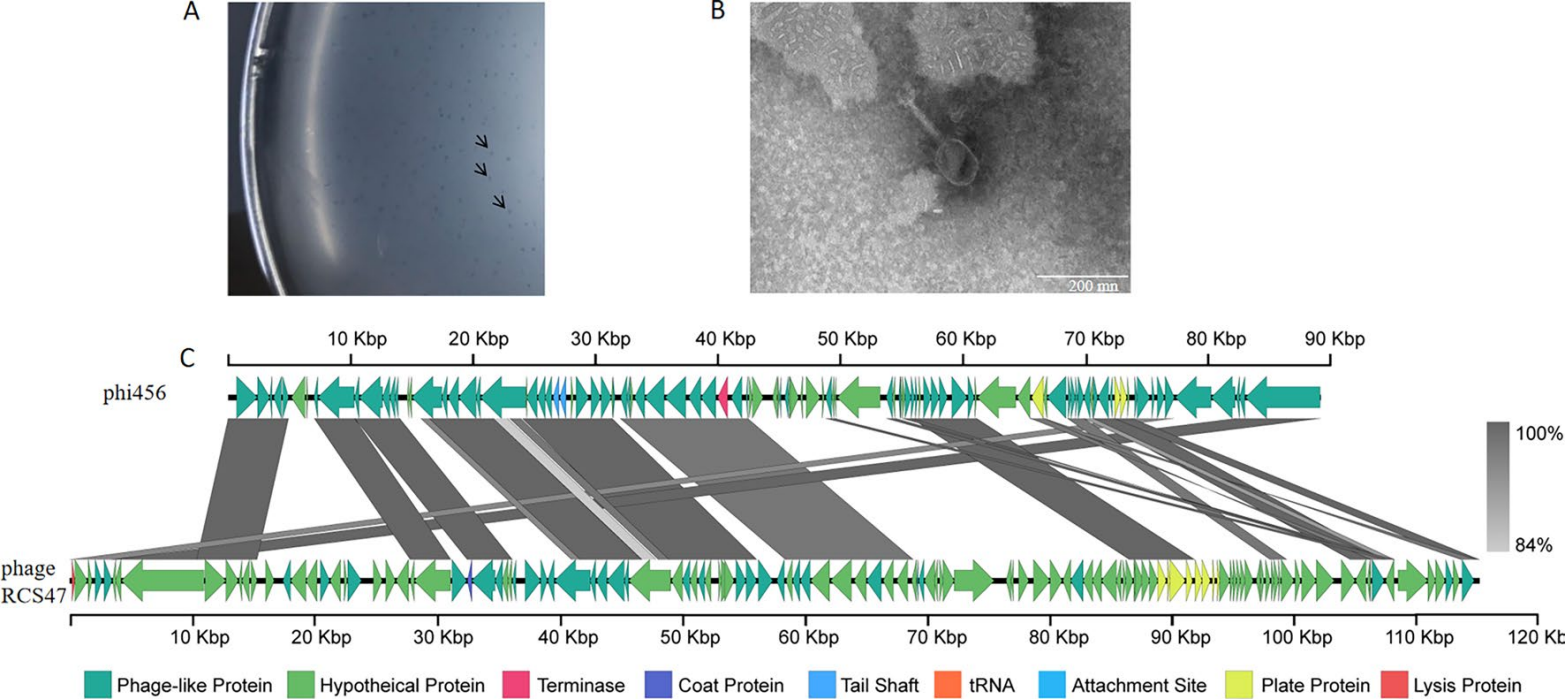
**Morphological characteristics and comparative genomic analysis of phi456.** **A** phi456 forms tiny and blurry plaques on MC1061 in the plaque assay, which are indicated by black arrows. **B** Transmission electron microscopy images of purified phage phi456. **C** The structure of prophage phi456. Phi456 showed a 70% nucleotide sequence identity with *Escherichia* phage RCS47. The unbroken line indicates the length of the sequence, while ORFs classified in the same functional categories are color-coded accordingly.

The genome size of prophage phi456 was determined to be 88.4 kb, and a total of 78 open reading frames were identified, including integrase and lysin. The transmission electron microscopy result revealed that phi456 belongs to the *Myoviridae* family (Figure 1A). By blasting on NCBI, phi456 showed a 70% nucleotide sequence identity with *Escherichia* phage RCS47 (Figure 1B).

In VirulenceFinder analyses, no virulence genes were detected in phi456. The reference sequences of 167 phages in NCBI were used to compare with phi456. Through genome multiple sequence alignment, the phylogenetic trees supported the assignment of phi456 to a sublineage shared by Myoviridae (Additional file 1), which was consistent with the results observed by TEM.

### The induction rate of phi456 increases at 42 ℃ or when interacting with cells

**Figure 2:**
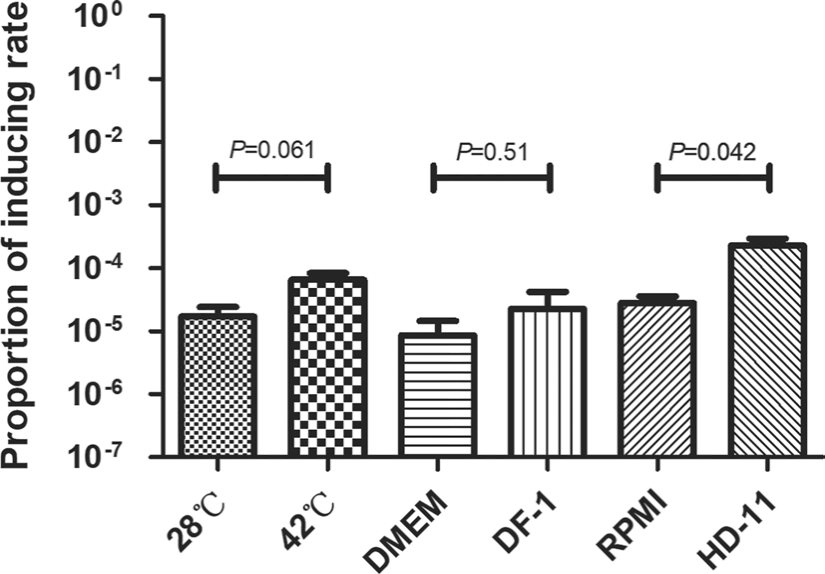
**Induction rate of prophage in different environments.** The number of bacterial genomes without phi456 was quantified by using primers flanking of phi456. The quantification of the target sequence was performed by normalizing to the reference gene *purA*. The error bars indicate standard deviations. Student’s *t*-test was used to analyse the data.

According to the environmental conditions in chickens, we determined the spontaneous induction rates under selected conditions. As shown in Figure 2, the results showed that the induction rate of phi456 at 42 ℃ was 3.8 times as high as that at 28 ℃. Meanwhile, the results of the cell experiment indicated that when DE456 was cultured with DF-1 or HD11 cells, the proportion of induction increased from 8.6 × 10^–6^ to 2.3 × 10^–5^ and 2.8 × 10^–5^ to 2.3 × 10^–4^, respectively. These data suggested that the higher temperatures and interaction with cells could promote the prophage phi456 in DE456 to enter the lytic state.

### Spontaneous prophage induction of phi456 enhances biofilm formation due to increasing eDNA

**Figure 3:**
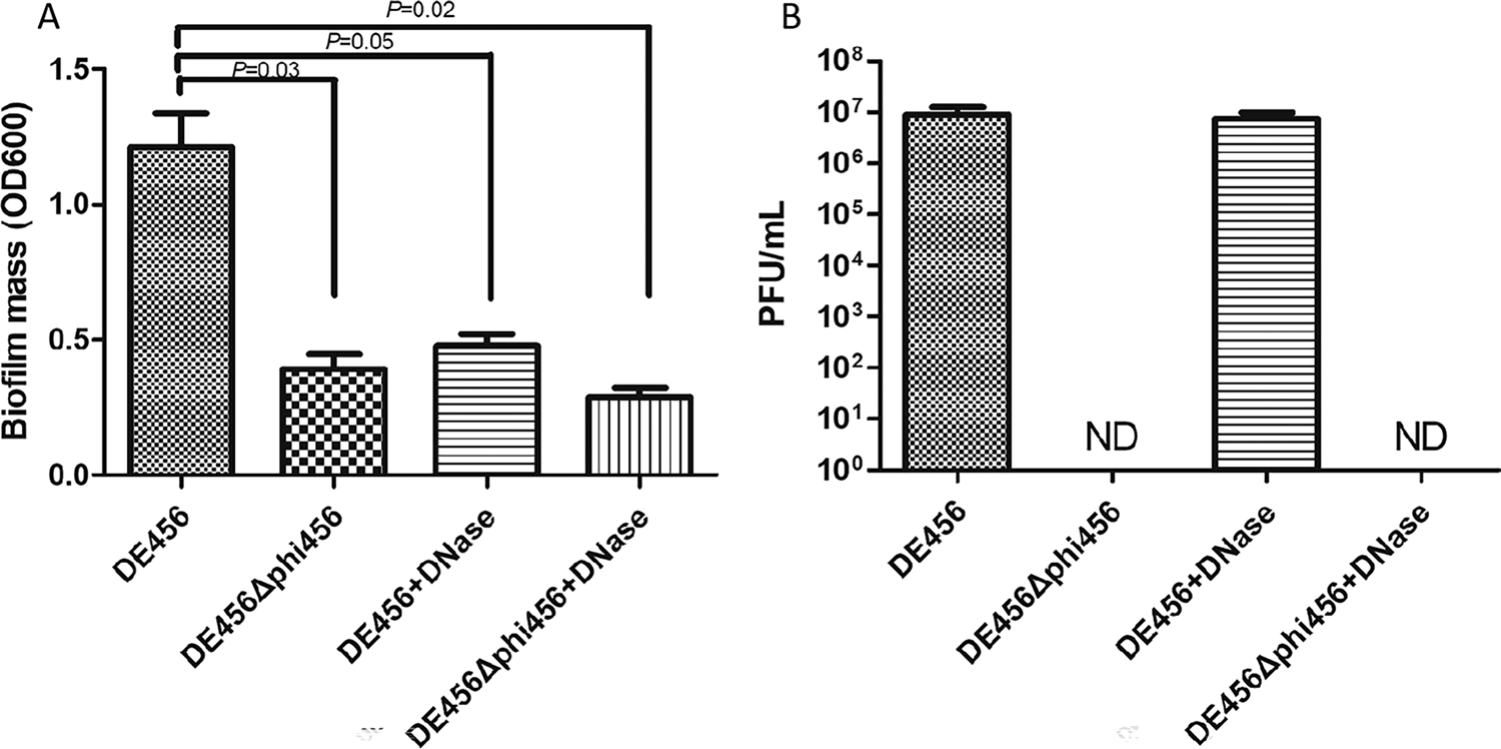
**Presence of prophage phi456 in bacterial genome increases biofilm formation.** **A** Biofilm formation of DE456 and DE456Δphi456 with or without DNase for 36 h. **B** The amount of phage particles phi456 during biofilm formation. The data represent the mean values obtained from six replicate wells in 96-well plates across three independent experiments. Error bars indicate standard deviations. Student’s *t*-test was used to analyse the data.

The effect of phi456 on its host cell’s ability to form biofilms was measured in 96-well microtiter plates. The WT strain had a significantly higher biofilm mass than DE456Δphi456 (Figure 3A). Meanwhile, nearly 10^7^ PFU/mL phage particles were released during biofilm formation by DE456 (Figure 3B). In the process of releasing prophages, the content of extracellular eDNA could be increased, which may contribute to the formation of biofilms. Thus, DNase was used to digest eDNA to test whether phi456 promotes biofilm formation by increasing eDNA. As shown in Figure 3A, after the addition of DNase, the amount of DE456 biofilm formation was similar to that of DE456Δphi456. However, the addition of DNase did not change the release of phage particles from DE456 during biofilm formation (Figure 3B). The results indicated that the promotion of phi456 in the process of DE456 biofilm formation is mainly due to the increase in eDNA.

### Deletion of phi456 decreases resistance to oxidative and acid stress

**Figure 4:**
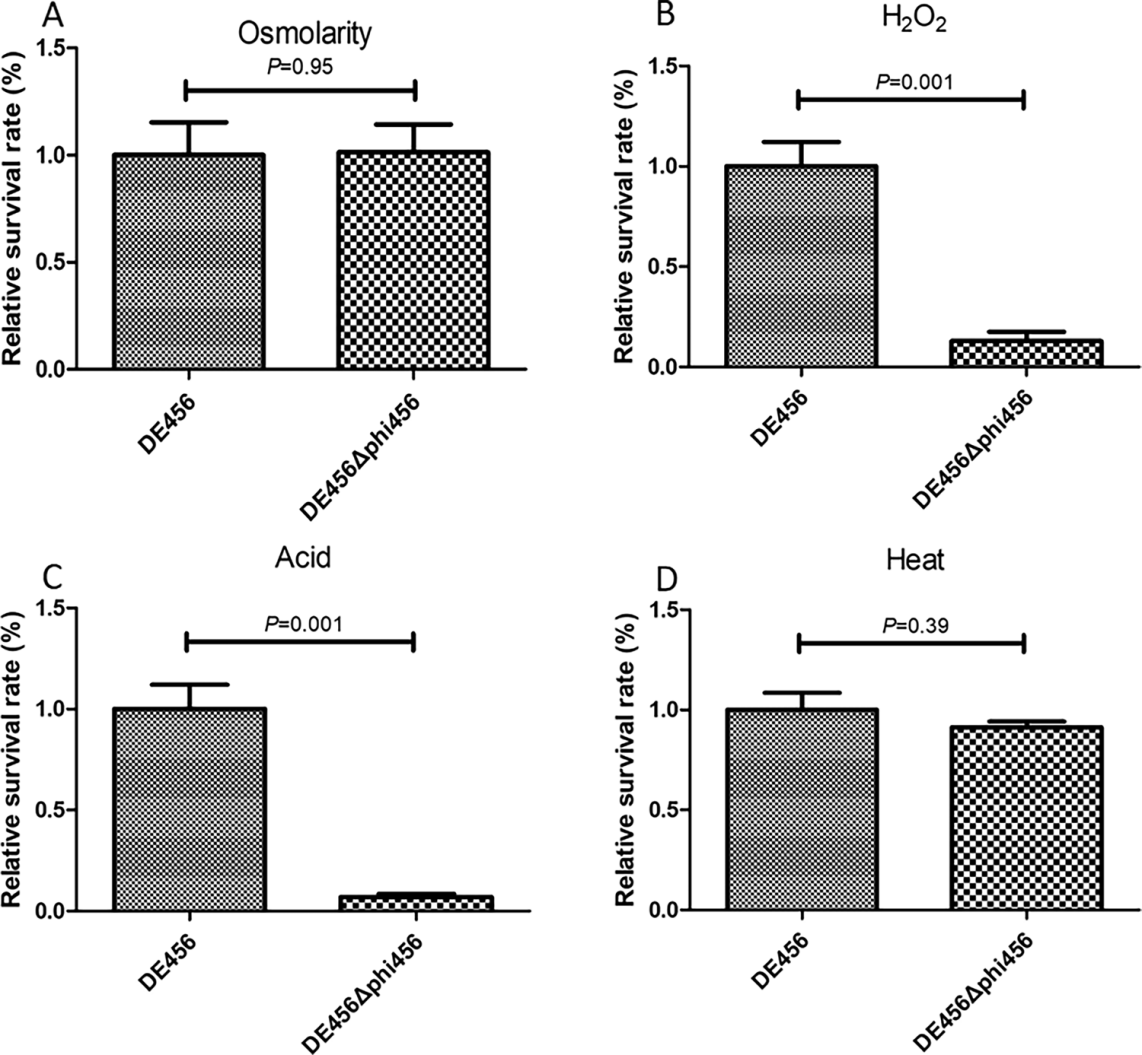
**The effect of prophage on stress-related phenotypes.** Survival for DE456 and DE456Δphi456 after challenging with **A** osmotic stress (2.4 M NaCl for 30 min), **B** oxidative stress (30 mM H_2_O_2_ for 15 min), **C** acid stress (pH 3 for 30 min) and **D** heat stress (65 °C for 10 min). Data are the mean of 3 replicates ± SD. Error bars indicate standard deviations. Student’s *t*-test was used to analyse the data.

To investigate the potential involvement of prophage phi456 in conferring stress resistance, the survival ability of the strains in adverse environments was assessed. Compared to the WT strain, the survival rate of the phi456 mutant strain was significantly decreased to 13% (*P* = 0.001, Figure 4B) and 7% (*P* = 0.001, Figure 4C) under oxidative stress (30 mM H_2_O_2_, 15 min) and acidic (pH 3, 30 min) conditions, respectively. However, the survival rates of DE456 and DE456Δphi456 were similar under osmotic stress (2.4 M NaCl, 30 min) and heat (65 °C, 10 min) (Figures 4A and D). Therefore, the presence of prophage phi456 contributed to the ability of the bacterial host to withstand oxidative and acid stress.

### Deletion of phi456 decreases colonization in vivo

**Figure 5:**
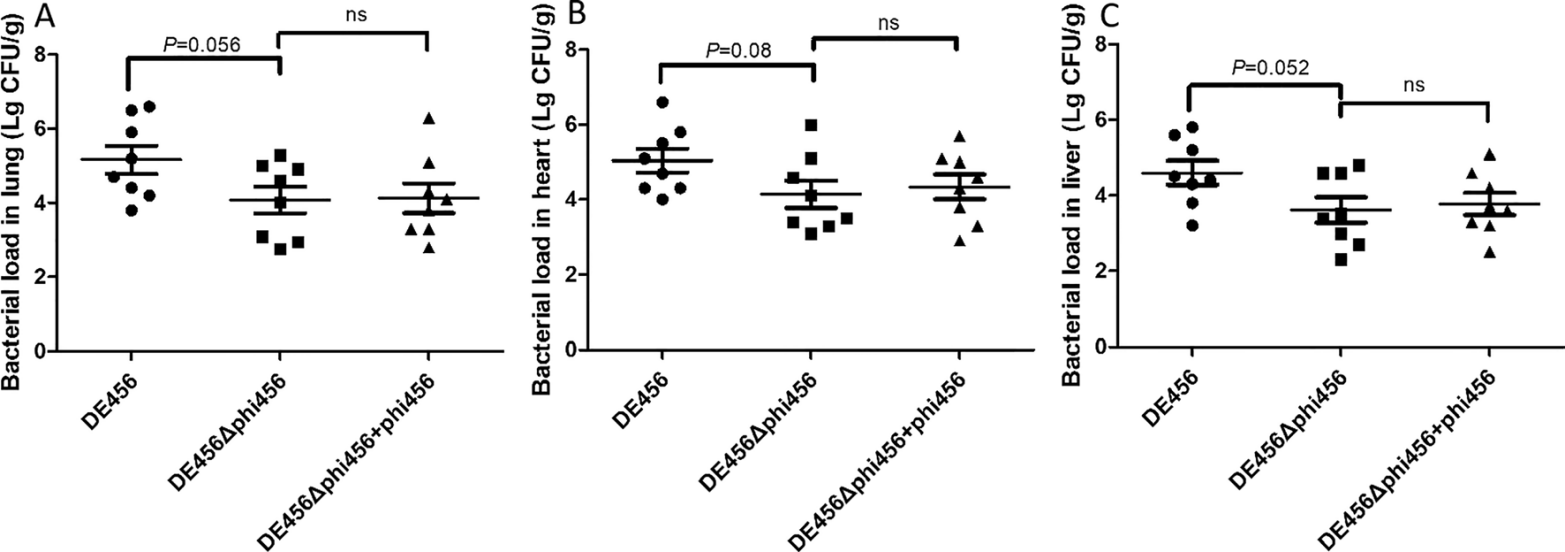
**Bacterial counts during infection in vivo.** Chickens were inoculated by the air sac route with strains or a bacteria-phage suspension, respectively. At 24 hpi, chickens were sacrificed and the bacterial load in **A** lung, **B** heart, and **C** liver was quantified. The data was subjected to analysis using the Mann–Whitney *U* test.

The post-infection bacterial counts of DE456 in the lung, heart, and liver were 1.6 × 10^5^ CFU/g, 1.0 × 10^5^ CFU/g, and 4.0 × 10^4^ CFU/g, respectively. However, the number of DE456Δphi456 in the corresponding organs was 1.1 × 104 CFU/g, 1.3 × 104 CFU/g, and 3.2 × 10^3^ CFU/g (Figure 5). Meanwhile, when DE456Δphi456 infected chickens, adding phage particles did not affect the bacterial loads in the organs.

**Figure 6:**
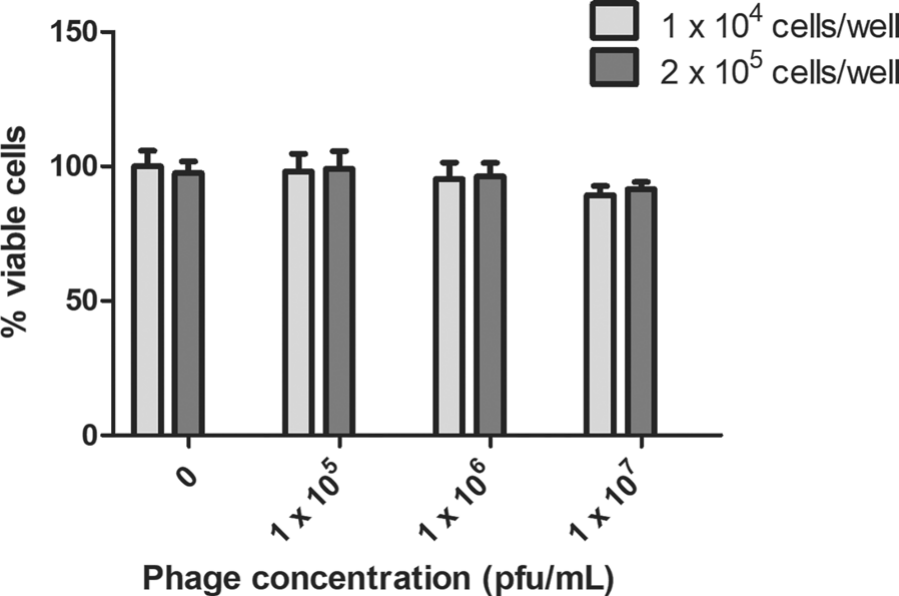
**Cytotoxicity of phage particles phi456.** After being exposed to phi456 in DMEM for 24 h, the viability of DF-1 chicken embryo fibroblast cells was assessed using the trypan blue exclusion assay. The data represented the average of three independent assays. Error bars indicate standard deviations. Student’s *t*-test was used to analyze the data.

To explore the impact of phi456 particles on cells, the phage cytotoxicity was measured by Trypan blue assays. As shown in Figure 6, there was no significant difference in cell viability under different multiplicities of infection. Additionally, the growth curve of DE456Δphi456 also showed no significant difference compared with that of DE456 in LB medium (Data not shown). The data indicated that phi456 may enhance colonization ability in vivo by regulating gene expression related to bacterial colonization in DE456.

### Deletion of phi456 leads to decreased type I fimbriae expression and adhesion ability

**Figure 7:**
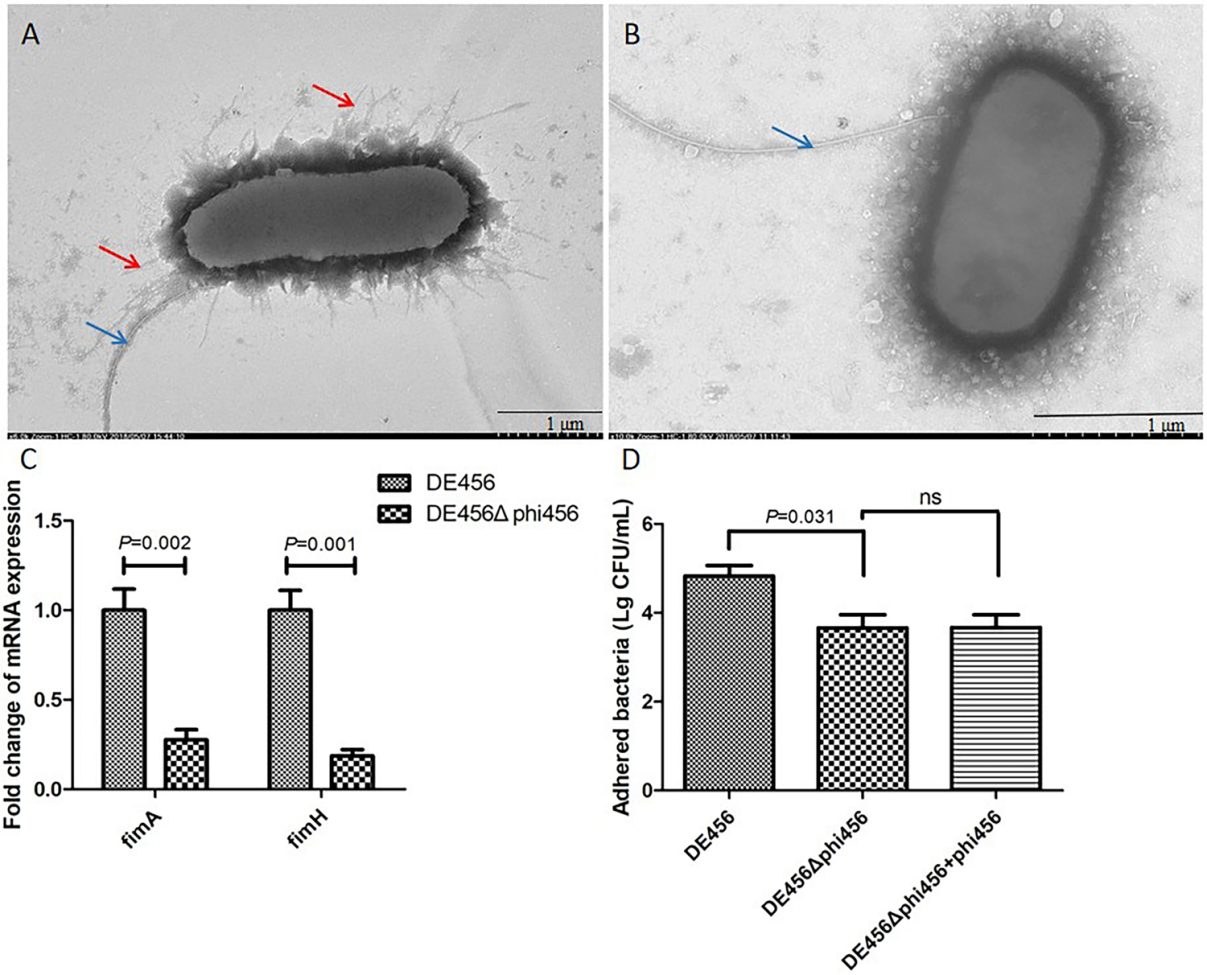
**Deletion of phi456 leads to decreased type I fimbriae and adhesion ability.** Transmission electron microscopy images of DE456 (**A**) and DE456Δphi456 (**B**). The red arrow points to the I fimbria, while the blue arrow points to the flagella. **C** Quantification of genes related to type I fimbriae by qRT-PCR. The relative expression level of *fimA* and *fimH* in the DE456Δphi456 was compared to WT. The quantification of the target gene was performed by normalizing to the reference gene *dnaE*. **D** Adhesion assay. Values are the average of three independent experiments. The error bars indicate standard deviations. Student’s *t*-test was used to analyse the data.

The cellular morphologies were observed via transmission electron microscopy, and a striking observation was that the deletion of phi456 led to a reduction in I fimbriae in DE456Δphi456 (Figures 7A, B). Furthermore, the transcription levels of *fimA* (subunit of type I fimbriae) and *fimH* (type I fimbriae apical adhesin) were measured by qRT‒PCR. The results showed that the transcription levels of these two genes in DE456Δphi456 were significantly reduced by 73% and 82%, respectively, compared to WT (Figure 7C). In the adhesion process, type I fimbriae plays an important role, so we evaluated the adhesion ability by a DF-1 cell model. After the same incubation time, the adhered bacteria of DE456Δphi456 was 4.6 × 10^3^ CFU, while the number of DE456 was 6.6 × 10^4^ CFU (*P* < 0.05) (Figure 7D).

### Deletion of phi456 decreases survival ability in HD11 cells

**Figure 8:**
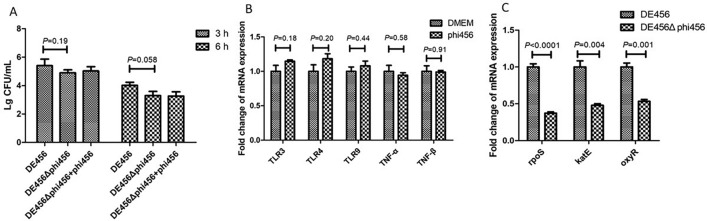
**Prophage phi456 genes influence survival ability in macrophage cells HD-11.** **A** The bacterial survival in HD-11 infected with DE456 or DE456Δphi456 at a MOI of 20 bacteria per cell. At 3 h or 6 hpi, the cells were lysed and bacterial counts were measured. **B** Quantification of genes related to toll-like receptors and tumor necrosis factor by qRT-PCR. The quantification of the target gene was performed by normalizing to the reference gene β-Actin. **C** Quantification of genes related to oxidative stress response by qRT-PCR. The relative expression level of *rpoS*, *katE* and *oxyR* in the DE456Δphi456 was compared to WT. The quantification of the target gene was performed by normalizing to the reference gene *dnaE*. The error bars indicate standard deviations. Student’s *t*-test was used to analyze the data.

The survival rates of DE456Δphi456 and DE456 were assessed in avian macrophages. The results showed that DE456 exhibited a higher survival rate than DE456Δphi456 in HD11 cells, with a three to fivefold increase in intracellular survival capacity (Figure 8A). However, the addition of phi456 did not affect the bacteria’s ability to survive in HD11. Furthermore, qRT-PCR results showed that the addition of phi456 did not change (*P* > 0.05) the transcriptional level of toll-liked receptors (TLR3, TLR4 and TLR9) and tumor necrosis factor (TNF-α and TNF-β) levels at the HD-11 cell level (Figure 8B). To further explore the reasons why knocking out phi456 lead to reduced survival ability in HD11 cells, some genes associated with resistance to oxidative stress were measured. The qRT-PCR results showed that the transcription levels of *rpoS*, *katE*, and *oxyR* in DE456Δphi456 were significantly decreased by 2–3 times after H_2_O_2_ treatment compared with WT (*P* < 0.05, Figure 8C). The data suggested that phi456 may enhance the survival ability of bacteria in macrophages by affecting the expression level of genes associated with oxidative stress resistance.

## Discussion

Infection caused by APEC strains results in significant loss for the global poultry industry [34]. Thus, improving our understanding of APEC’s pathogenic mechanisms would be immensely advantageous to the poultry industry, facilitating the management of APEC-related illnesses. A previous study in our lab found that the prophage in DE456 could be released during infection [7]. Genome analysis revealed that phi456 belongs to the *Myoviridae* family and shares high homology with *Escherichia* phage RCS47 (Figure 1). It has been reported that phage RCS47 belongs to the P1 phage and is associated with the spread of resistance genes through lysogenic infection [33]. Our goal in this study was to investigate whether prophage phi456 plays a role in bacterial fitness and pathogenesis.

Bacteria are capable of withstanding various adverse environments by forming biofilms [35]. In our study, the biofilm formed by DE456Δphi456 was thinner than that of the WT strain. There are two main mechanisms by which prophages affect biofilm formation: one is the release of eDNA through lysogenic conversion [29], and the other is affecting the genes related to biofilm formation [36]. Because phi456 had a high spontaneous rate in vitro, we also found that nearly 10^7^ PFU/mL phage particles were released during biofilm formation by DE456 (Figure 3B). Thus, we speculate that phi456 in DE456 promotes biofilm formation by increasing eDNA. The addition of DNase did not change the induction rate of phi456 during biofilm formation but reduced biofilm formation of DE456 to the level of DE456Δphi456 (Figure 3A), which confirms what we suspected.

After encountering physical barriers, bacterial pathogens must overcome the host's innate defense mechanisms to cause disease [37]. In this regard, the ability to survive within macrophages provides pathogenic *E. coli* a competitive advantage. In our study, DE456 exhibited a higher survival rate than DE456Δphi456 in HD11 cells, with a three to five increase in its intracellular survival capacity (Figure 8A). It was previously demonstrated that some bacteriophages produced by lysogenic strains could activate Toll-like receptors and inhibit tumor necrosis factor, which impairs bacterial clearance [38, 39]. However, adding phi456 phage particles did not result in significant changes in the transcriptional levels of toll-like receptors and tumor necrosis factors at the HD-11 cell level (Figure 8B), nor did it affect survival rates (Figure 8A). In addition to directly killing microorganisms through proteases or peptides, macrophages also kill bacteria by producing a large number of reactive oxygen species [40]. Thus, the ability of bacteria to resist oxidative stress was thought to be associated with the viability of macrophages. In our study, the survival rate of the phi456 mutant strain was decreased to 13% (*P* = 0.001, Figure 4B) under oxidative stress (30 mM H_2_O_2_, 15 min). Although the specific mechanism is unclear, this may explain to some extent why the survival ability of DE456Δphi456 in HD11 is decreased. Given that *rpoS* serves as the critical regulator of the stationary phase stress response [41] and positively modulates the expression of genes essential for the oxidative stress response, such as *katE* encoding catalase and *oxyR* encoding a regulatory protein sensor for oxidative stress [42], we investigated the effect of phi456 deletion on the transcriptional activity of *rpoS*, *katE* and *oxyR*. The results showed that compared with the WT strain, the transcription levels of *rpoS*, *katE* and *oxyR* in DE456Δphi456 were decreased by 2–3 times after H_2_O_2_ treatment for 15 min (Figure 8B). Single-cell analysis revealed that *lfgB*, a cryptic prophage protease, protects *Escherichia coli* from oxidative stress by cleaving the antitoxin MqsA to derepress *rpoS* [43]. In addition, some prophages have been found to encode superoxide dismutase [44], an enzyme that catalyzes the conversion of superoxide into hydrogen peroxide and oxygen, thereby enhancing the bacterium's ability to withstand oxidative stress. In this study, in order to find the genes that may play the above roles in phi456, we conducted knock-out verification of dozens of genes in phi456 (data not shown). Unfortunately, we have not found the key genes that affect oxidative stress. Although the specific mechanism still needs further study, these findings suggested that phi456 is related to the resistance to oxidative.

Adhesion is a crucial step in APEC infection and plays a pivotal role in determining the pathogenic variances among bacterial isolates [45]. The adhesion experiment results showed that the adhesion ability of DE456Δphi456 was decreased tenfold compared to that of the WT strain (Figure 7D). To explore the mechanism by which phi456 affects adhesion and colonization ability, we first observed the cell morphology by TEM. We were surprised that the deletion of phi456 led to a reduction in type I fimbriae (Figures 7A and B). FimH and FimA participate in the biosynthesis of type I fimbriae. Specifically, FimH functions as a receptor-specific adhesin for type I fimbriae [46], while FimA is a subunit of this structure[47]. We further detected the transcription levels of genes related to the biosynthesis of type I fimbriae, and the results showed that the mRNA levels of *fimA* and *fimH* in DE456Δphi456 were three- and five-fold lower than those in WT (Figure 7C). Including *E. coli*, type I fimbriae facilitate bacterial adherence to the host cell mannose receptor and assist the bacteria in colonization in vivo. Moreover, no genes associated with virulence were detected in phi456, and phi456 did not exhibit cytotoxic effects on cells (Figure 6). Hence, we speculat that phi456 contributes to the adhesion ability of DE456 by affecting the formation of type I fimbriae.

In summary, this study highlights that the prophage phi456 plays a crucial role in helping bacterial hosts to survive in unfavorable conditions and enhancing the colonization ability in DE456. However, which genes in phi456 may play a key role in regulating bacterial biology and its effect on other APEC strains needs to be further explored. Although phi456 does not carry virulence genes, it can regulate type I fimbrial formation and the expression of genes related to oxidative stress in DE456, so it is worth noting that phi456 is a temperate phage that might be able to integrate into the genome of particular host bacteria and form a prophage, which may increase their fitness in adverse environments and enhance virulence.

### Supplementary Information

**Additional file 1. Phylogenetic analysis tree.** Based on the genome multiple sequence alignment neighbor-joining tree analysis supported the assignment of phi456 to a sublineage shared by Myoviridae.

## Data Availability

The datasets generated and/or analysed during the current study are available from the corresponding author upon reasonable request.
