# Supplementary figures and images for: The spontaneously produced lysogenic prophage phi456 promotes bacterial resistance to adverse environments and enhances the colonization ability of avian pathogenic Escherichia coli strain DE456
Source: Vet Res. 2024 Mar 26;55:37. doi: 10.1186/s13567-024-01292-z (PMC10967188; doi:10.1186/s13567-024-01292-z)

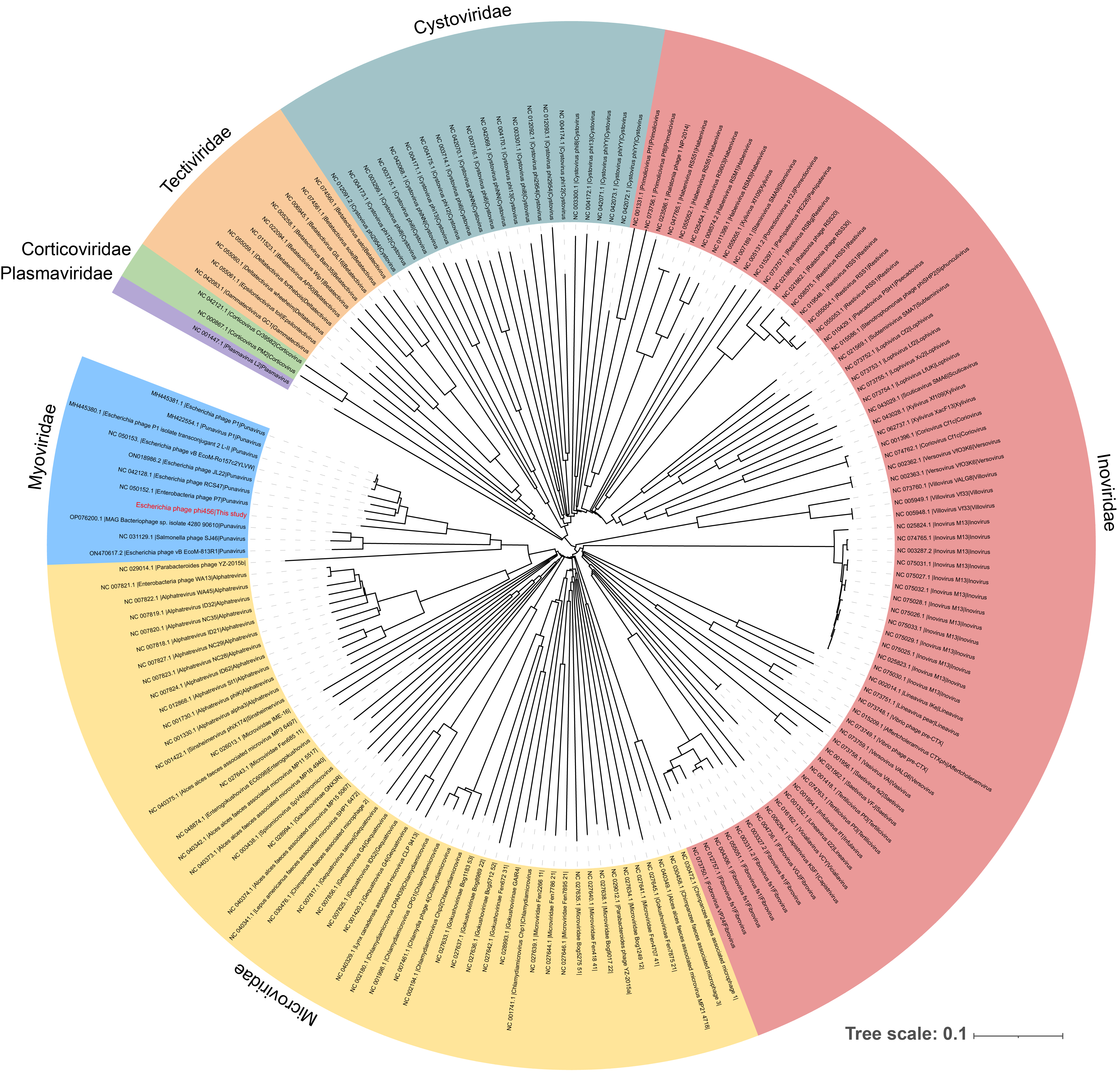

Supplement: Supplementary file 1 — Additional file 1. Phylogenetic analysis tree. Based on the genome multiple sequence alignment neighbor-joining tree analysis supported the assignment of phi456 to a sublineage shared by Myoviridae. [file 13567_2024_1292_MOESM1_ESM.jpg]
